# Population Pharmacokinetic Modeling of Piperacillin/Tazobactam in Healthy Adults and Exploration of Optimal Dosing Strategies

**DOI:** 10.3390/ph18081124

**Published:** 2025-07-27

**Authors:** Yun Jung Lee, Gaeun Kang, Dae Young Zang, Dong Hwan Lee

**Affiliations:** 1 Department of Physical Medicine and Rehabilitation, Myongji Hospital, Goyang 10475, Republic of Korea; yjlee@mjh.or.kr; 2 Division of Clinical Pharmacology, Chonnam National University Hospital, Gwangju 61469, Republic of Korea; bp00092@cnuh.com; 3 Division of Hematology-Oncology, Department of Internal Medicine, Hallym University Sacred Heart Hospital, Hallym University College of Medicine, Anyang 14066, Republic of Korea; fhdzang@hallym.or.kr; 4 Department of Clinical Pharmacology, Hallum University Sacred Heart Hospital, Hallym University College of Medicine, Anyang 14066, Republic of Korea

**Keywords:** piperacillin, tazobactam, population pharmacokinetics, Monte Carlo simulation, healthy, adult

## Abstract

**Background/Objectives:** Current dosing recommendations for piperacillin/tazobactam suggest adjustments only for patients with creatinine clearance (CrCl) below 40 mL/min, potentially neglecting the variability in drug exposure among patients with a CrCl greater than 40 mL/min. This study aimed to develop a population pharmacokinetic (PK) model for piperacillin/tazobactam and explore optimal dosage regimens tailored by renal function and pathogen susceptibility. **Methods**: Twelve healthy adults received a single intravenous dose of piperacillin/tazobactam (4 g/0.5 g). Population PK models were developed using nonlinear mixed-effects modeling. Monte Carlo simulations were conducted to identify optimal dosing regimens across various renal functions and MIC levels, guided by pharmacodynamic targets defined as the percentage of time that free drug concentrations exceed the minimum inhibitory concentration (*f*T_>MIC_). **Results**: PK profiles of both drugs were best described by two-compartment models. Estimated glomerular filtration rate (eGFR) adjusted by body surface area and body weight were identified as significant covariates influencing drug clearance and peripheral volume of distribution. Simulations showed that the standard dosing regimen (4/0.5 g q6h with 30 min infusion) achieved a 90% probability of target attainment (PTA) for 50%*f*T_>MIC_ at MIC values up to 4 mg/L in patients with normal renal function. However, this regimen often did not achieve a 90% PTA for stringent targets (100%*f*T_>MIC_, 100%*f*T_>4MIC_) or higher MICs, particularly in patients with eGFR ≥ 130 mL/min. **Conclusions**: These findings suggest current dosing regimens may be inadequate and highlight the potential of alternative strategies, such as extended or continuous infusion, which warrant further investigation in clinical populations to optimize therapeutic outcomes.

## 1. Introduction

Piperacillin/tazobactam is a penicillin-class β-lactam/β-lactamase inhibitor combination that has received approval from the U.S. Food and Drug Administration (FDA) and the European Medicines Agency (EMA) for the treatment of a wide range of bacterial infections, including intra-abdominal infections, skin and skin structure infections, urinary tract infections, female pelvic infections, nosocomial pneumonia, and community-acquired pneumonia [1,2]. It exhibits broad-spectrum antibacterial activity against aerobic and anaerobic Gram-positive and Gram-negative bacteria. It is frequently used for empirical or definitive treatment of critically ill patients with sepsis [3,4,5].

Early clinical data have affirmed the efficacy of this fixed-dose combination (at a dose ratio of 8:1) for managing moderate to severe polymicrobial infections, consistent with its in vitro activity [6]. Johnson and colleagues examined its pharmacokinetics (PK) in patients with renal disease and found that the half-life and area under the plasma drug concentration-time curve of piperacillin and tazobactam concomitantly increase as creatinine clearance (CrCl) decreases. Based on these findings, they recommended lower daily doses for patients with a CrCl below 40 mL/min [7]. In clinical practice, such as in the management of adults with nosocomial pneumonia, piperacillin/tazobactam is typically prescribed at a fixed dose of 4/0.5 g q6h for patients with a CrCl greater than 40 mL/min [1,2]. However, this fixed regimen does not account for the variability of renal function beyond this threshold, which may result in significant differences in drug exposure. These differences may affect pharmacodynamic (PD) outcomes and efficacy, increasing the risk of resistance. Critically ill patients, such as those with sepsis, often experience pathophysiological changes like fluid shifts from leaky capillaries and altered protein binding, which complicate drug disposition and dosing [8]. These shifts affect the volume of distribution (Vd) and drug clearance (CL). Decreased renal function can reduce CL, while an increase in cardiac output may enhance it. Given these complexities, model-informed precision dosing should be employed to account for individual pathophysiological conditions, including decreased, normal, or increased renal function.

Previous studies have shown that the therapeutic efficacy of piperacillin/tazobactam administered at a fixed dose of 4/0.5 g q6h can vary based on renal function, particularly in patients with a CrCl exceeding 40 mL/min. Efficacy was evaluated by the probability of target attainment (PTA), defined as the total percentage of a 24 h period during which the concentration of the free (unbound to protein) drug surpasses the minimum inhibitory concentration (MIC) under steady-state conditions (*f*T_>MIC_) [9,10,11]. Asín-Prieto et al. established a population PK model for critically ill patients undergoing continuous renal replacement therapy, in which piperacillin/tazobactam was administered q6h over 20 min [12]. Their simulations were aimed at achieving a 90% PTA of 100%*f*T_>MIC_ and indicated that with a CrCl of 50 mL/min, the breakpoints were 8 mg/L, which decreased to 2 mg/L as the CrCl increased to 100 mL/min. Sime et al. developed a PK model for patients with febrile neutropenia [13]. Using this model to simulate a fixed-dose administration over 30 min, they aimed for a 90% PTA of 50%*f*T_>MIC_ and found that at a CrCl of 40 mL/min, the breakpoints were at 16 mg/L, which decreased to 4 mg/L at 80 mL/min and further to 1 mg/L at 120 mL/min. Ishihara et al. developed a PK model for elderly patients with pneumonia and conducted simulations targeting a 90% PTA of 50%*f*T_>MIC_ with a 1 h infusion q6h [14]. The results indicated that at a CrCl of 50 mL/min, the breakpoints were at 16 mg/L, but were reduced to 8 mg/L when CrCl increased to 60 mL/min.

While the preceding studies highlight the need for dosing optimization, they were conducted in clinically complex patient populations where it is difficult to isolate the effect of renal function from other disease-related confounding factors. This necessitates a foundational study in healthy adults to establish a PK baseline free from disease-related confounding factors. Moreover, as existing PK data from healthy volunteers originate primarily from Western populations, and ethnic differences in drug disposition are well-recognized, a population-specific model for healthy Korean adults was required to serve as a more appropriate reference. This study was therefore designed to address this dual knowledge gap.

The objectives of this study were to develop a population PK model for piperacillin/tazobactam in healthy adults and to perform Monte Carlo simulations using this model to suggest optimal dosing and administration strategies for adult patients with a CrCl of 40 mL/min or greater.

## 2. Results

### 2.1. Participants

The demographic and clinical characteristics of the 12 healthy adult subjects are listed in Table 1. All baseline laboratory values were within normal clinical ranges, confirming their healthy status. The median body weight of our Korean subjects was 61.7 kg, and key renal function metrics such as CrCl and estimated glomerular filtration rates (eGFR) confirmed normal renal function across the cohort. During the stepwise covariate analysis, various forms of the CKD-EPI equation utilizing creatinine, cystatin C, or both were evaluated as potential covariates for clearance [15]. As these estimators yielded broadly consistent values in our population, only representative variables with potential clinical relevance were included in Table 1. All participants completed the study without any adverse drug reactions.

### 2.2. Population Pharmacokinetic Analysis

**Figure 1:**
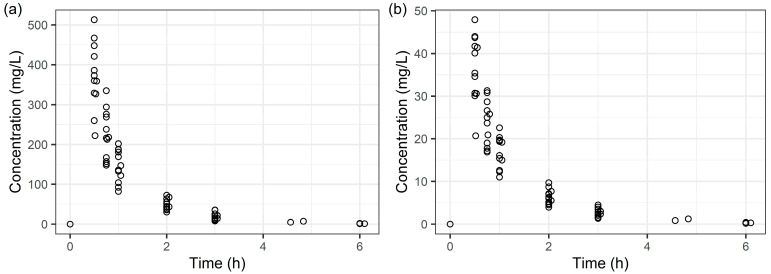
Piperacillin (**a**) and tazobactam (**b**) concentration–time profile in healthy adults. The open circles denote the observed plasma concentrations.

A total of 84 plasma samples (Figure 1) were used to establish a population PK model for piperacillin/tazobactam. The concentration-time profiles for both piperacillin and tazobactam were best described by a two-compartment model. The structural PK parameters for the two-compartment model were CL, Vd for the central compartment (V1), Vd for the peripheral compartment (V2), and intercompartmental CL between V1 and V2 (Q), as shown in Table 2 and Table 3. The BSA-adjusted eGFR, calculated using the CKD-EPI equations and creatinine levels, was identified as a significant covariate for CL in the final PK models for both piperacillin and tazobactam (Table 2 and Table 3). In addition, body weight was a significant covariate for the V2 in these models. For piperacillin, lean body mass (LBM) was also a significant covariate for Q in the final PK model. The incorporation of eGFR as a covariate on CL significantly improved the model fit for piperacillin; its removal from the final model increased the OFV by 16.414 and reduced the associated interindividual variability (IIV) from 13.2% to 7.17%. Similarly, the inclusion of CrCl on CL was significant for tazobactam, increasing OFV by 13.318 upon removal and reducing IIV from 17.6% to 6.95%. Details of the covariate selection process are provided in Appendix A (Table A1 and Table A2). During covariate evaluation, WT was significantly associated with V2 for both piperacillin and tazobactam, with notable reductions in OFV. In contrast, the inclusion of WT as a covariate on V1 did not yield statistically significant improvements. In the final PK model for tazobactam, IIV on the V1 was initially tested; however, due to its high relative standard error (RSE > 70%) and negligible contribution to model fit improvement, this parameter was excluded from the final model. Conversely, for piperacillin, the IIV on V1 showed acceptable precision (RSE: 28.7%, shrinkage: 19.1%) and was retained in the final model (Table 2 and Table 3).

**Figure 2:**
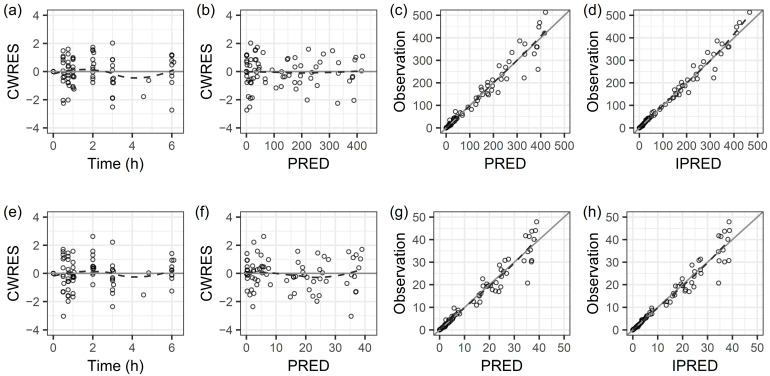
Goodness-of-fit plots for the final pharmacokinetic model of piperacillin (upper) and tazobactam (lower): (**a**,**e**) conditional weighted residuals (CWRES) vs. time, (**b**,**f**) CWRES vs. population predicted concentration (PRED) (**c**,**g**) observed concentration vs. PRED and (**d**,**h**) observed concentration vs. individual predicted concentration (IPRED). The dashed lines represent smoothing spline curves.

**Figure A1:**
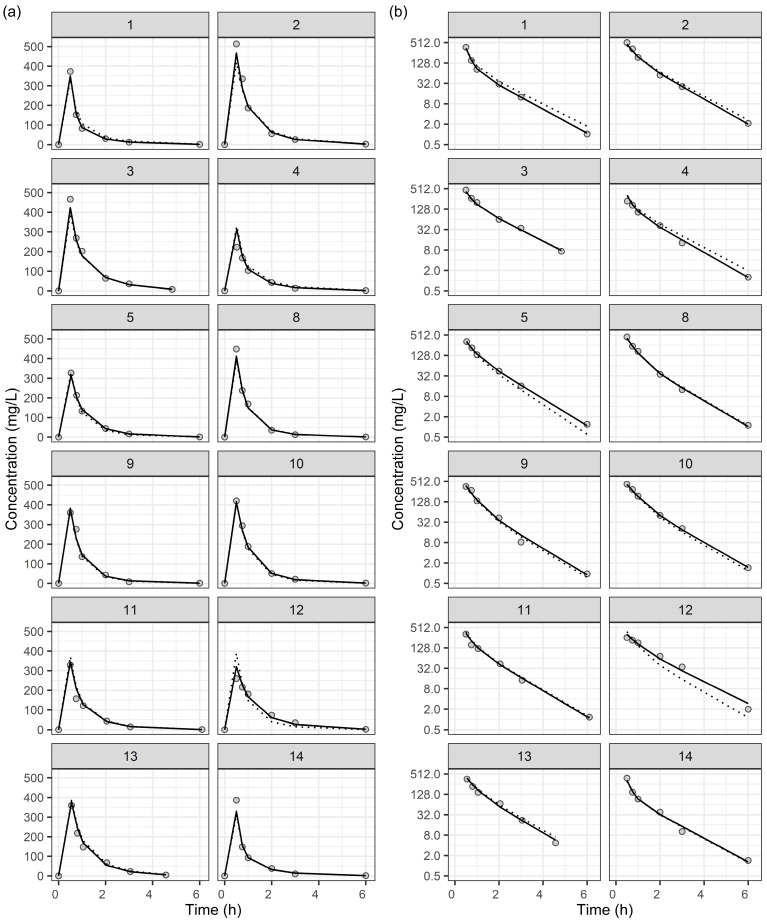
Individual fit plots for piperacillin (**a**) normal scale, (**b**) semi-log scale: closed circles, observed concentrations; solid line, individual predicted concentrations; dotted line, population predicted concentrations. The numbers in each panel correspond to the individual subject number.

**Figure A2:**
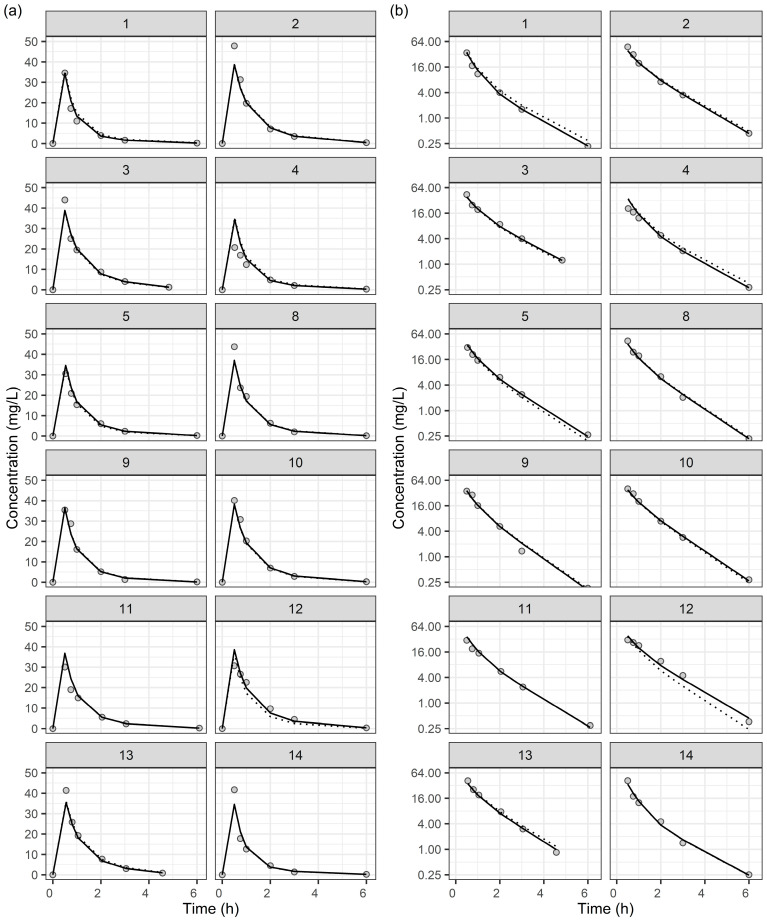
Individual fit plots for tazobactam (**a**) normal scale, (**b**) semi-log scale: closed circles, observed concentrations; solid line, individual predicted concentrations; dotted line, population predicted concentrations. The numbers in each panel correspond to the individual subject number.

**Figure A3:**
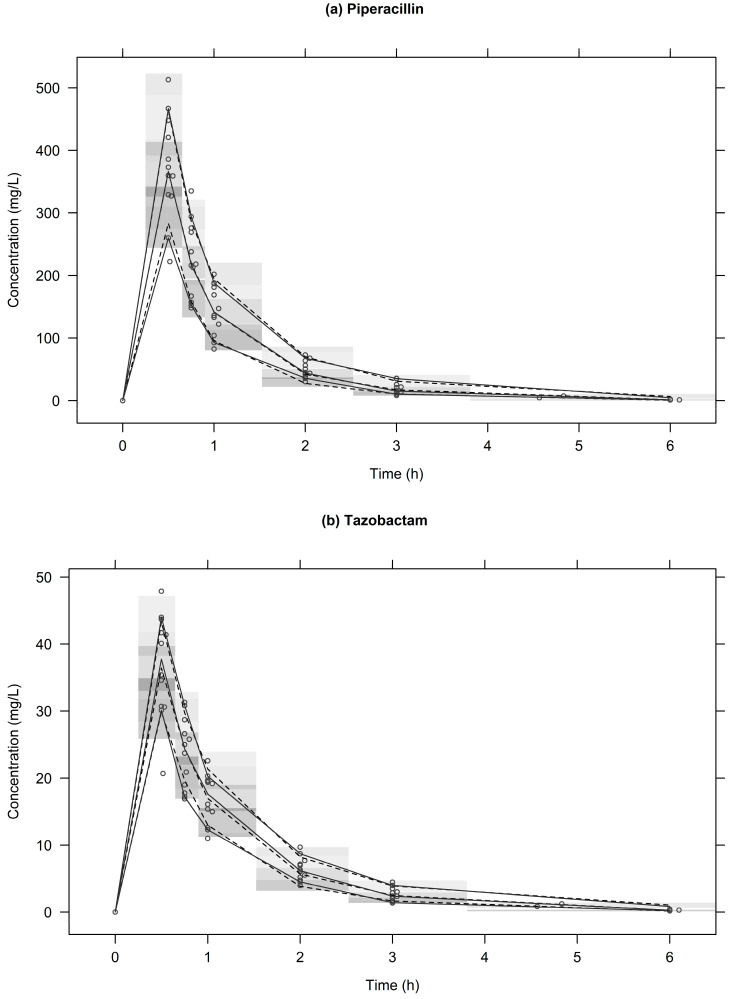
Visual predictive check from simulated concentrations of 1000 virtual datasets of piperacillin (**a**) and tazobactam (**b**): closed circles, observed concentrations; solid lines, 10th, 50th, and 90th percentiles of observations; dashed lines, 10th, 50th, and 90th percentiles of simulated concentrations. The shaded areas represent the 95% confidence intervals for the simulated 10th (darkest grey), 50th (medium grey), and 90th (lightest grey) percentiles.

Figure 2 presents diagnostic goodness-of-fit plots for the final PK model of piperacillin and tazobactam. Plots of conditional weighted residuals (CWRES) versus time and versus population predicted concentrations showed largely uniform around the x-axis or the line of identity (y = x), suggesting that the structural models were correctly adjusted and without bias. Minor deviations for two data points around 4–5 h in panels (a) and (e) were observed, but the overall distribution of CWRES near zero indicated an acceptable model fit. The time courses for the individual fit plots are shown in Figure A1 and Figure A2 for piperacillin and tazobactam, respectively. The visual predictive checks (VPC) for piperacillin and tazobactam are shown in Figure A3. The measured 10th, 50th, and 90th percentiles fell within the 95% confidence intervals (CI) of the respective simulated percentiles, indicating that the final PK models adequately described the observed concentration variability and had good predictive performance.

### 2.3. Dosage Simulation

**Figure 3:**
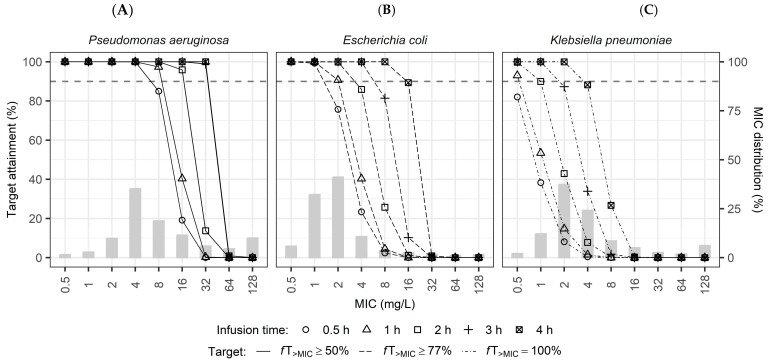
Probability of target attainment (PTA) versus MIC for the empirical piperacillin/tazobactam regimen (4 g/0.5 g q6h) in virtual subjects with normal renal function. Panels show PTA for different infusion durations (0.5, 1, 2, 3, and 4 h) against (**A**) *Pseudomonas aeruginosa* (Target: 50%*f*T_>MIC_), (**B**) *Escherichia coli* (Target: 77%*f*T_>MIC_), and (**C**) *Klebsiella pneumoniae* (Target: 100%*f*T_>MIC_). Gray bars represent the MIC frequency distribution for each pathogen. Horizontal dashed lines denote the 90% PTA threshold.

Simulation results for empirical dosing in subjects with normal renal function showed variable PTA across the different infusion durations and targeted pathogens (Figure 3). For clinical context, according to the European Committee on Antimicrobial Susceptibility Testing (EUCAST) database [16], the clinical breakpoint for susceptibility is an MIC ≤ 8 mg/L for *Enterobacterales* (including *Escherichia coli* and *Klebsiella pneumoniae*) and an MIC ≤ 16 mg/L for *Pseudomonas aeruginosa*.

**Figure A4:**
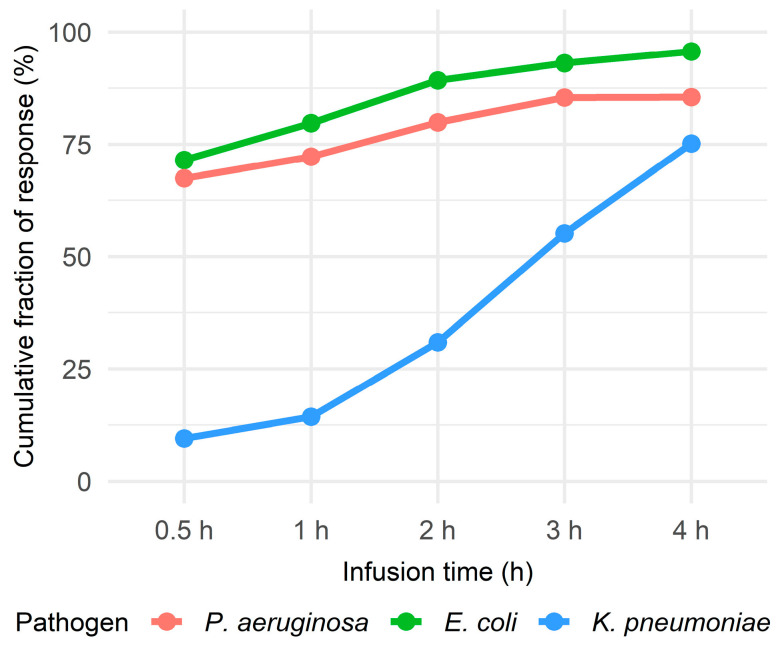
Cumulative fraction of response (%) according to infusion time across pathogens, based on the probability of achieving predefined pharmacodynamic targets: 50%*f*T_>MIC_ for *P. aeruginosa*, 77%*f*T_>MIC_ for *E. coli*, and 100%*f*T_>MIC_ for *K. pneumoniae*.

The standard dosing regimen of piperacillin/tazobactam (4 g/0.5 g q6h with a 0.5 h infusion) achieved adequate PTA (>90%) at lower MIC values. However, a pronounced decline in PTA was observed at elevated MIC values, particularly for *E. coli* and *K. pneumoniae*, even with prolonged infusion durations. For *P. aeruginosa*, extending the infusion duration to ≥3 h significantly improved PTA, maintaining PTA > 90% up to an MIC of 16 mg/L. In contrast, for all three pathogens, shorter infusion times (≤1 h) consistently failed to achieve the 90% PTA threshold at MIC values of 8 mg/L or higher. These results underscore the limitations of current label-based empirical dosing regimens against pathogens with higher MIC values and highlight the potential advantage of extended infusion strategies. As shown in Figure A4, the cumulative fraction of response (CFR) increased with longer infusion times across all pathogens evaluated. The improvement was most pronounced in *K. pneumoniae*, where CFR rose from approximately 15% at 0.5 h to over 75% at 4 h. In contrast, *E. coli* and *P. aeruginosa* exhibited higher baseline CFRs (>65% at 0.5 h), and their response curves showed a more gradual ascent, reaching near-maximal levels (>90%) at 4 h.

**Figure 4:**
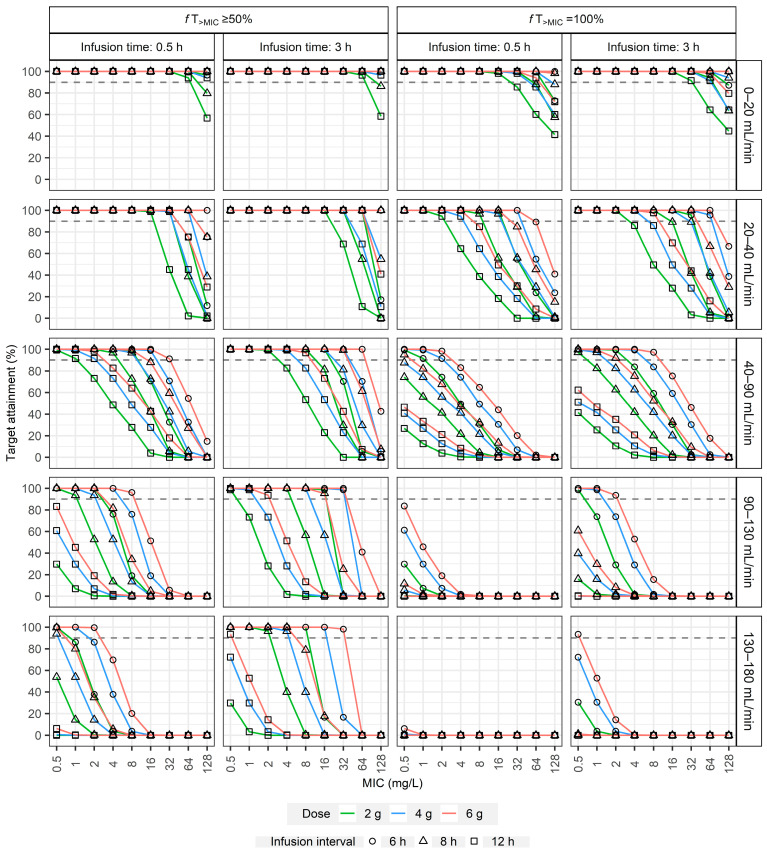
Probability of target attainment (PTA) versus MIC for targeted intermittent piperacillin/tazobactam regimens achieving 50%*f*T_>MIC_ or 100%*f*T_>MIC_ across various renal function categories (eGFR). Panels compare PTA for different doses (2 g, 4 g, or 6 g) and dosing intervals (6 h, 8 h, or 12 h) with 0.5 h versus 3 h infusion durations. Horizontal dashed lines denote the 90% PTA threshold.

**Figure 5:**
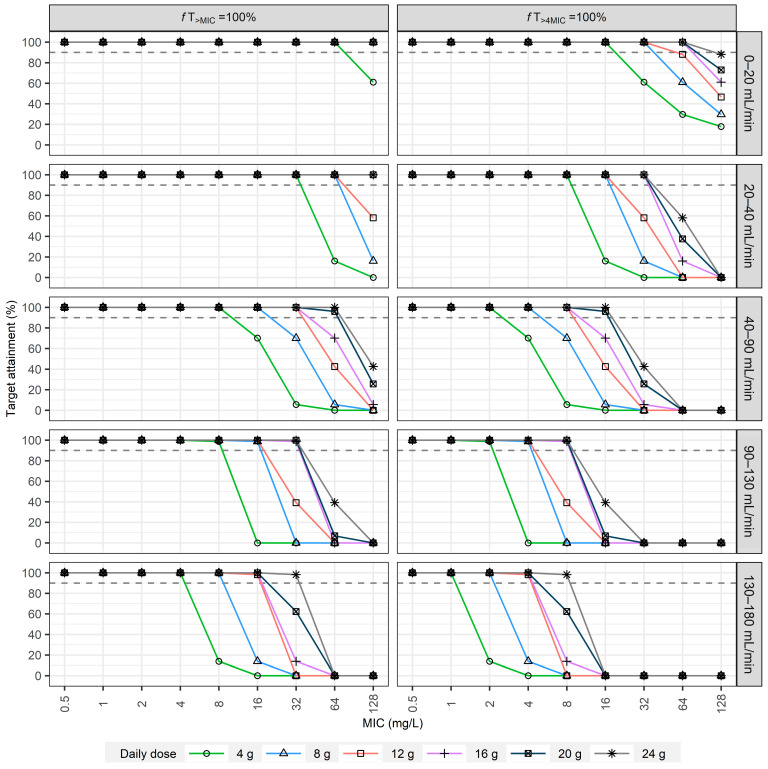
Probability of target attainment (PTA) for continuous infusion regimens of piperacillin across different renal function categories. PTA values were evaluated for achieving pharmacodynamic targets of 100%*f*T_>MIC_ or 100%*f*T_>4MIC_ at various MIC levels (0.5–128 mg/L). Daily doses ranged from 4 to 24 g/day. Horizontal dashed lines denote the 90% PTA threshold.

**Figure A5:**
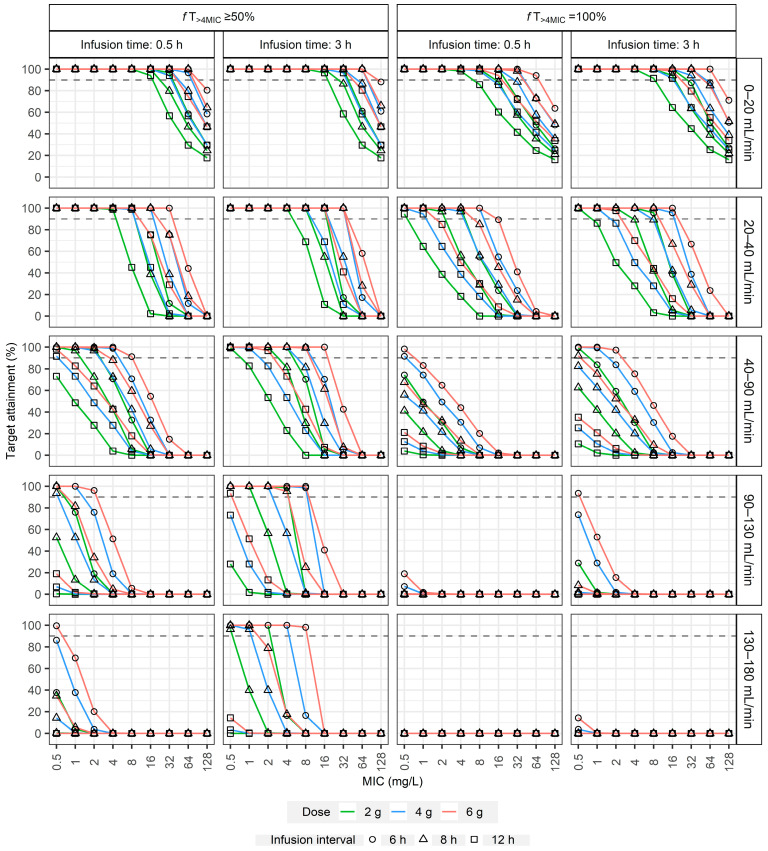
Probability of target attainment (PTA) versus MIC for targeted intermittent piperacillin/tazobactam regimens achieving 50%*f*T_>4MIC_ or 100%*f*T_>4MIC_ across various renal function categories (eGFR). Panels compare PTA for various dosing regimens with 0.5 h versus 3 h infusion durations. Horizontal dashed lines denote the 90% PTA threshold.

The second simulation study provided PTA results for targeted dosing regimens across various renal function groups, infusion strategies, and MIC values (Figure 4, Figure A5 and Figure 5). For less stringent PD targets (50%*f*T_>MIC_ and 50%*f*T_>4MIC_), shorter infusions (0.5 h) were generally sufficient to achieve adequate PTA at lower MIC values. However, their effectiveness diminished substantially at higher MICs, a challenge particularly pronounced in subjects with eGFR ≥ 130 mL/min, where even high doses often failed to achieve 90% PTA for MICs ≥ 4 mg/L (Figure 4 and Figure A5). Extending the infusion duration from 0.5 h to 3 h consistently enhanced PTA across all renal function categories, especially at moderate MICs.

Achieving the stricter PD targets (100%*f*T_>MIC_ and 100%*f*T_>4MIC_) was considerably more challenging. For patients with normal renal function (e.g., eGFR 90–130 mL/min), high-dose intermittent regimens, such as 6 g q6h infused over 3 h, were required to reliably cover MICs up to only 2 mg/L for the 100%*f*T_>MIC_ target (Figure 4). In contrast, continuous infusion strategies demonstrated superior performance; a daily dose of 16 g via continuous infusion achieved nearly 100% PTA for the same target up to an MIC of 16 mg/L (Figure 5). Table A3 and Table A4 summarize the recommended dosing regimens derived from these simulations. Collectively, these results reinforce the need for personalized, model-informed dosing strategies based on renal function and pathogen susceptibility to optimize therapeutic outcomes.

To aid in understanding our results, a user-friendly Shiny app has been developed (available at https://dhlee.shinyapps.io/pthv/).

## 3. Discussion

This study addresses a notable gap in the literature by conducting a population PK analysis of piperacillin/tazobactam in healthy Korean adults, providing foundational insights for future clinical applications. Our population PK analysis demonstrated that the PK of both piperacillin and tazobactam were best described by two-compartment models. The final models incorporated several significant covariates: for both drugs, renal function (eGFR, calculated by the CKD-EPI formula) influenced CL and WT affected V2, and LBM was specifically identified as a key determinant of Q for piperacillin.

Although several renal function estimators were evaluated, including creatinine-based, cystatin C-based, and combined equations, the final selection of the creatinine-based CKD-EPI equation was driven by statistical performance in the model. Given the limited sample size, it is plausible that different renal function markers might have emerged as significant in a larger or more heterogeneous cohort. The statistically supported assignment of WT specifically to V2 rather than V1 might appear somewhat counterintuitive. Typically, V1 is more directly associated with body size metrics, reflecting the initial distribution space of the drug. However, given the hydrophilic properties of piperacillin and tazobactam, the peripheral compartment likely reflects distribution into extravascular spaces such as interstitial fluids, muscle, and skin tissues, volumes strongly correlated with body weight. Thus, this data-driven covariate assignment appears physiologically plausible. The stepwise covariate modeling provided strong support for including LBM as a covariate on Q for piperacillin (ΔOFV = 14.798), a finding that, while not typical, was statistically robust. Initially, theory-based allometric scaling [17] involving total body weight or LBM across all PK parameters was attempted, but these broader approaches did not improve model performance. A mechanistic rationale is that LBM closely reflects lean tissues and extracellular fluid compartments, significantly relevant to hydrophilic antibiotics like piperacillin. Nonetheless, these findings, particularly the novel association of LBM with Q, must be interpreted with caution. Although the parameter was statistically robust and passed bootstrap validation, the limited sample size creates an inherent risk of overfitting. Therefore, this specific finding requires external validation in larger, more diverse populations before its clinical significance can be established.

In this study, typical values for piperacillin CL and steady-state volume of distribution (Vss = V1 + V2) were 11.2 L/h and 8.83 L, respectively, corresponding to 0.174 L/h/kg and 0.148 L/kg when normalized by body weight. These findings are slightly lower for clearance and notably lower for Vss compared with previously reported noncompartmental analyses in healthy adults. For example, Bryson and Brogden summarized piperacillin’s CL and Vss as approximately 14.5 L/h (0.181 L/h/kg) and 15.0 L (0.188 L/kg), respectively, assuming an average weight of 80 kg [6]. Similarly, Daniel and Krop reported CL and Vss values of 15.5 L/h (0.194 L/h/kg) and 16.7 L (0.209 L/kg), respectively, under the same assumption [18].

Population PK studies have reported a range of CL and Vss values. Bulitta et al. compared the PK of piperacillin in 8 Caucasian patients with cystic fibrosis to that in 26 healthy volunteers. In their study, the CL for the healthy individuals was 10.4 L/h (0.146 L/h/kg), and the Vss was 11.8 L (0.166 L/kg) [19]. Bulitta et al. conducted another study on 4 healthy Caucasian adults. In their study, the CL was 10.1 L/h (0.144 L/h/kg), and the Vss was 12.7 L (0.181 L/kg) [20]. Felton et al. developed a PK model based on data from 53 critically ill patients and 25 healthy volunteers. In their study, the CL for healthy individuals was 11.45 L/h (0.142 L/h/kg), and the Vss was 8.18 L (0.102 L/kg) [21]. It is also important to consider the demographic differences between these cohorts, particularly body weight. The median body weight of our Korean subjects was 61.7 kg, which is considerably lower than the mean or median body weights of the healthy Caucasian volunteers in the studies by Bulitta et al. (71.1 kg and 77.5 kg, respectively) and Felton et al. (81 kg) [19,20,21]. Notably, despite nearly 20 kg lower median body weight compared to the subjects in the Felton et al. study, the Vss was remarkably similar (8.99 L vs. 8.18 L). This suggests that factors beyond body weight, such as body composition or ethnicity, may significantly influence piperacillin’s Vd.

In patients with normal or above-normal renal function, the Vss was generally higher than in our study. This variation is further illustrated by population PK studies on critically ill sepsis patients. Roberts et al. found a median CL and Vss for piperacillin at 17.1 L/h (0.214 L/h/kg) and 25 L (0.313 L/kg), respectively [22]. Similarly, Udy et al. reported a median CrCl of 122 mL/min and a median weight of 88.4 kg, with CL and Vss of 16.3 L/h (0.184 L/h/kg) and 38.7 L (0.438 L/kg), respectively [23]. Sime et al. reported on a population PK study involving critically ill sepsis patients with a median CrCl of 94 mL/min/1.73 m^2^ and a median weight of 78 kg, where CL and Vss for piperacillin were 18.0 L/h (0.231 L/h/kg) and 18.3 L (0.235 L/kg), respectively [13].

Differences in Vss are also observed in studies involving Asian populations, suggesting factors beyond ethnicity, such as clinical status, play a significant role. Direct comparisons are challenging, but various population PK studies illustrate this point. For example, Kim et al. conducted a study on critically ill Korean patients with a median CrCl of 53.1 mL/min and a median weight of 54 kg. They reported a clearance of 5.05 L/h (0.0904 L/h/kg) and a Vss of 22.8 L (0.422 L/kg) [24]. Ishihara et al. studied elderly Japanese patients with an average CrCl of 38.0 mL/min and an average weight of 45.5 kg. They reported CL and Vss values of 4.58 L/h (0.101 L/h/kg) and 12.4 L (0.271 L/kg), respectively [14]. Chen et al. evaluated patients with nosocomial infections who had an average CrCl of 68.7 mL/min and an average weight of 61.1 kg. The CL and Vss were 9.14 L/h (0.150 L/h/kg) and 12.2 L (0.200 L/kg), respectively [25].

Synthesizing these comparisons reveals the central contribution of our work. The Vss in our healthy Korean subjects was markedly lower than that reported in patient populations, which was often two- to four-fold greater. This stark difference, likely driven by disease-state pathophysiology, underscores the necessity of establishing an “uncontaminated” baseline from healthy individuals to accurately quantify the effects of illness on drug disposition. Moreover, while other healthy-volunteer studies exist, they predominantly feature Caucasian populations. As ethnic differences can influence PK, our study addresses a critical gap by establishing the first population PK model specifically in healthy Korean adults. This work therefore provides a dual contribution: a clean baseline free from disease-related confounders and a population-specific foundation essential for developing tailored therapeutic strategies for Korean and East Asian patients.

The adequacy of current piperacillin/tazobactam dosing recommendations for patients with CrCl ≥ 40 mL/min was further examined through simulations, along with an evaluation of alternative dosing regimens stratified by narrower renal function intervals. In addition to the most used PK/PD target (50%*f*T_>MIC_), simulations examined more stringent targets suitable for severe infections, including 50%*f*T_>4MIC_, 100%*f*T_>MIC_, and 100%*f*T_>4MIC_. The target of 50%*f*T_>MIC_ originated from efficacy observations in mouse models infected with Streptococcus pneumoniae and patients with otitis media caused by *S. pneumoniae* and *Haemophilus influenzae* [9]. This target is recognized as a critical determinant of clinical success [26,27,28]. Additional research in ICU populations demonstrated that higher *f*T_>MIC_ correlates with improved bacteriological and clinical outcomes [29], promoting the adoption of these more rigorous thresholds in multiple studies [24,30,31,32].

The observed superiority of extended and continuous infusion strategies in our simulations is directly explained by the time-dependent pharmacodynamic nature of piperacillin. As a β-lactam antibiotic, its bactericidal activity is not primarily dependent on achieving high peak concentrations but rather on the cumulative percentage of time the free drug concentration remains above the MIC during the dosing interval [9,10]. Standard short infusions generate high peaks, but drug concentrations decline rapidly, thus shortening the duration of effective exposure. In contrast, extended infusion lowers the peak concentration but prolongs the time above the MIC, thereby maximizing the %*f*T_>MIC_ for a given daily dose. Continuous infusion represents the ultimate application of this principle by maintaining a constant drug concentration, which explains its superior ability to achieve stringent targets (e.g., 100% *f*T_>MIC_), as demonstrated in Figure 5.

The first simulation assessed FDA and EMA dosing recommendations for piperacillin/tazobactam using population PK modeling and Monte Carlo simulations. Results showed that the standard regimen (4 g/0.5 g q6h, 0.5 h infusion) achieved adequate PTA (≥90%) at lower MICs for common nosocomial pneumonia pathogens (Figure 3). However, at elevated MIC levels, especially with *E. coli* and *K. pneumoniae*, significant reductions in PTA were observed even when infusion durations were prolonged. Conversely, extended infusions (≥3 h) notably improved PTA for *P. aeruginosa*, maintaining adequate attainment at MICs up to 16 mg/L. These findings highlight potential limitations of the current standard dosing approach and underscore the value of extended infusion durations, particularly against pathogens with higher MIC values. This trend was further supported by the CFR analysis (Figure A4), which demonstrated that prolonged infusion times enhanced the cumulative probability of achieving pharmacodynamic targets across all pathogens. Notably, *K. pneumoniae* showed the greatest increase in CFR—from approximately 15% at 0.5 h to over 75% at 4 h—largely due to the more stringent PD target applied (100%*f*T_>MIC_). In contrast, *E. coli* and *P. aeruginosa*, for which lower targets of 77% and 50% *f*T_>MIC_ were used respectively, exhibited higher CFRs even at shorter infusion durations. These differences emphasize that CFR outcomes are influenced not only by infusion strategy but also by the choice of PD target, which in this study varied across pathogens by design.

In the second dosing simulations, both intermittent and continuous infusion regimens were evaluated across a wide range of renal function categories (Figure 4, Figure 5 and Figure A5). While shorter infusions (0.5 h) were generally sufficient for less stringent PD targets (50%*f*T_>MIC_ and 50%*f*T_>4MIC_) at lower MIC values, their effectiveness diminished substantially at higher MICs, especially in the presence of augmented renal clearance (ARC) [33], a condition characterized by an eGFR of 130 mL/min or higher (Figure 4 and Figure A5). Extending infusion durations to 3 h significantly enhanced PTA across all renal function categories, especially at intermediate MIC values. Achieving more stringent PD targets (100%*f*T_>MIC_ and 100%*f*T_>4MIC_) required higher dosing and longer infusion durations, especially for patients with eGFR ≥ 40 mL/min.

Continuous infusion regimens consistently provided higher PTA compared to intermittent infusions across all renal function groups and MIC values (Figure 5). These findings emphasize the clinical importance of individualized dosing strategies based on renal function and pathogen susceptibility, supporting the adoption of model-informed precision dosing approaches to optimize therapeutic efficacy and patient outcomes. These simulation data provide quantitative support for refining current clinical dosing practices of piperacillin/tazobactam, potentially improving therapeutic outcomes in critically ill patients with nosocomial infections.

Our study’s PK/PD breakpoints (the highest MIC at which the target PTA was achieved) showed significant differences compared to other studies that applied similar dosing regimens to patients with comparable renal functions. For a PK/PD index of 50%*f*T_>MIC_, we observed that patients with normal renal function could achieve a PTA of ≥90% up to an MIC of 4 mg/L with a 4 g q6h 0.5 h infusion, and up to an MIC of 32 mg/L with a 4 g q6h 3 h or 4 h extended infusion (Figure 3). Conversely, Patel et al. noted that for patients with a eGFR of 100 mL/min, the treatment goal was met up to an MIC of 1 mg/L with a 0.5 h infusion, and up to an MIC of 8 mg/L with a 4 h extended infusion [26]. In our assessment for a PK/PD index of 100%*f*T_>MIC_ in patients with an eGFR of 90–130 mL/min, a 0.5 h infusion of 4 g q6h did not meet the treatment goal at an MIC of 0.5 mg/L. However, extending the infusion to 3 h allowed achievement of the goal up to an MIC of 2 mg/L (Figure 4), while a 16 g/day continuous infusion met the goal up to an MIC of 32 mg/L (Figure 5). Asín-Prieto et al. found that in patients with a CrCl of 100 mL/min, the treatment goals were achieved up to a MIC of 2 mg/L with a 4 g q6h 20 min infusion, up to 8 mg/L with a 4 h extended infusion, and up to 16 mg/L with a 16 g/day continuous infusion [12]. Klastrup et al. also reported that in patients with a CrCl of 80–130 mL/min, the treatment goal was achieved up to a MIC of 16 mg/L with a 16 g/day continuous infusion [34]. In our study, the relatively low Vd observed in our healthy subjects likely contributes to the challenge of maintaining concentrations above MIC for 100% of the interval with intermittent infusions, resulting in lower PK/PD breakpoints for this stringent target due to a shorter half-life associated with the relatively lower Vd observed in healthy subjects compared to patients with a larger Vd. These findings underscore key limitations of the current standard dosing regimen, underscoring the potential advantages of extended infusion durations, especially for pathogens with elevated MIC values.

This study had several limitations. First, the relatively small sample size of 12 healthy adults, despite employing a rich sampling strategy, limited our ability to precisely estimate IIV and directly generalize findings to diverse patient populations, particularly the critically ill. Second, the narrow range of baseline renal function within our healthy adult participants significantly restricts the clinical applicability of the PK models and dosing recommendations, underscoring the need for validation in broader populations with impaired or augmented renal clearance. Third, the unbound fraction of piperacillin was assumed to be a fixed value of 0.7, which does not account for the considerable variability observed in patient populations, especially among the critically ill [35,36]. While such variability could theoretically influence target attainment, previous sensitivity analyses have demonstrated that the PTA for piperacillin remains robust to moderate fluctuations in protein binding [37]. This supports the validity of our approach despite this simplifying assumption. Finally, during the second simulation, lean body mass and body weight were held constant at median values. While this simplified interpretation by clearly isolating renal function effects, it may limit the generalizability to a more diverse patient population. Therefore, our simulation results represent a foundational PK baseline rather than directly applicable clinical dosing guidelines, highlighting the necessity for dedicated PK studies in patient populations exhibiting relevant pathophysiological changes.

In conclusion, this study demonstrated that current label-recommended dosing regimens for piperacillin/tazobactam may fail to provide sufficient therapeutic exposure in patients with eGFR ≥ 40 mL/min, particularly those with ARC. A population PK model incorporating eGFR, LBM, and body weight effectively predicted piperacillin/tazobactam PK in healthy adults. Monte Carlo simulations demonstrated that individualized dosing strategies tailored by detailed renal function stratification significantly enhance the probability of achieving target PK/PD indices. Therefore, to enhance clinical efficacy and minimize the risk of resistance, clinicians should consider integrating renal function-based, model-informed precision dosing strategies, including prolonged or continuous infusion for patients with eGFR ≥ 40 mL/min, into clinical practice. Although the simulation results suggest that higher dosing regimens could enhance the probability of achieving PD targets, such intensified regimens inherently carry risks of toxicity and adverse effects. Patients with borderline renal function or significant comorbidities might be particularly susceptible. Therefore, any clinical application of these findings must carefully balance efficacy with safety, incorporating therapeutic drug monitoring and rigorous clinical surveillance. Ultimately, validation through targeted PK studies in actual patient populations, especially critically ill patients, remains essential to refine these preliminary dosing insights and ensure their safe and effective clinical translation.

To build upon these foundational findings and address the limitations of this study, future research should proceed in several key directions. First, large-scale population PK studies in diverse patient populations—including critically ill Korean and East Asian patients with conditions such as sepsis or nosocomial pneumonia—are needed to quantify the impact of disease-related pathophysiology on drug disposition. Second, these studies should include participants across the full spectrum of renal function, from severe impairment to augmented clearance, to validate and refine the proposed dosing regimens. Third, direct measurement of unbound piperacillin concentrations should be incorporated to account for variability in protein binding, particularly in patients with hypoalbuminemia. Ultimately, prospective, randomized controlled trials are essential to evaluate the safety and efficacy of extended or continuous infusions compared to standard dosing and to confirm their clinical and microbiological benefits.

## 4. Materials and Methods

### 4.1. Participants

The study received approval from the Institutional Review Board at Hallym University Sacred Heart Hospital (IRB No. 2022-08-006, approved on 25 October 2022) and was conducted in compliance with the Declaration of Helsinki. This study was registered with the Clinical Research Information Service, operated by the National Institute of Health of the Korea Disease Control and Prevention Agency. The registration number is KCT0009855. More information can be found on the website: https://cris.nih.go.kr/cris/. The study was conducted in January 2023 at the Clinical Trial Center of the same institution. The inclusion criteria included: (1) Individuals aged between 19 and 55 at the time of screening; (2) Individuals free from congenital or chronic health conditions as confirmed by a medical evaluation; and (3) Individuals deemed eligible after comprehensive health screenings that encompassed medical history reviews, vital signs checks, physical examinations, hematological and biochemical blood tests, urinalysis, and other diagnostic tests. The exclusion criteria were: (1) Individuals with significant medical conditions affecting various systems, such as gastrointestinal, cardiovascular, respiratory, endocrine, hepatobiliary, hematologic-oncologic, musculoskeletal, renal, neurological, psychiatric, immunological, urological, ophthalmological, otolaryngological, or genetic disorders; (2) Individuals with past health issues that might influence the PK of the drugs, including liver or kidney diseases; (3) Individuals allergic to piperacillin/tazobactam or who have experienced adverse reactions to it; (4) Individuals testing positive for hepatitis B surface antigen, hepatitis C virus antibodies, HIV antigen or antibodies, or syphilis; and (5) Women who are pregnant, breastfeeding, or who may become pregnant.

### 4.2. Study Design

Eligible participants received an intravenous infusion of 4/0.5 mg piperacillin/tazobactam, prepared in 100 mL of saline solution and administered over 30 min. Venous blood samples (6 mL) were collected into tubes containing EDTA at predetermined time points to assess the PK characteristics. The planned sampling times were immediately before dosing and 0.5, 0.75, 1, 2, 3, and 6 h after beginning the infusion. Plasma concentrations of piperacillin/tazobactam were measured using a validated liquid chromatography-tandem mass spectrometry assay [24].

### 4.3. Population PK Analysis

The PK parameters for piperacillin/tazobactam were evaluated using nonlinear mixed-effects models with the assistance of NONMEM software (version 7.5, ICON Clinical Research LLC, North Wales, PA, USA). The first-order conditional estimation with interaction method was used for parameter estimation and incorporated both fixed and random effects. For the PK modeling of piperacillin/tazobactam, one-, two-, and three-compartment models were evaluated. Each model operated under first-order kinetics, except in the case of zero-order infusion processes. The models used the formula θ_i_ = θ × exp(η_i_) to define each parameter, where θ is the typical value, θ_i_ is the individual parameter value, and η_i_ represents the IIV, which is assumed to follow a normal distribution with zero mean and variance ω^2^. Residual variability, characterized by a normal distribution with zero mean and variance σ^2^, was assessed using additive, proportional, and combined additive-proportional error models. During population PK model development, covariance structures between key PK parameters (e.g., CL, V1, V2, Q) were evaluated. However, incorporating covariance terms did not significantly improve the model fit, as assessed by changes in OFV) and thus were not retained in the final model structures. Model refinement and selection processes were directed by variations in NONMEM OFVs, accuracy of parameter estimates indicated by relative standard errors, goodness-of-fit plots, VPC, and bootstrap analyses. Improvements in model structures were validated by decreases in OFV greater than 3.84 (for a single degree of freedom χ^2^ test) or 5.99 (for two degrees of freedom), which was considered statistically significant at *p* < 0.05. Model validation was conducted using four types of goodness-of-fit plots, including CWRES versus time, CWRES versus population predictions (PRED), observed concentrations versus PRED, and observed concentrations versus individual predictions (IPRED).VPC demonstrated that the observed concentrations of piperacillin/tazobactam were within 80% of the predicted intervals from 1000 model simulations. The variability in the final model’s predictions was examined through the medians and 95% confidence intervals derived from 2000 bootstrap samples. Significant covariates affecting PK parameters were determined through a stepwise forward selection and backward elimination process, with thresholds for inclusion at *p* < 0.01 (ΔOFV < −6.635) and exclusion at *p* < 0.001 (ΔOFV < 10.83). The covariate analysis included demographic factors such as gender, age, height, weight, LBM, body mass index, and body surface area, as well as biochemical parameters like serum protein, albumin, creatinine, and cystatin C levels. The impact of renal function on the elimination of piperacillin/tazobactam was assessed using the Cockcroft-Gault [38], Modification of Diet in Renal Disease [39], and Chronic Kidney Disease Epidemiology [15] equations. The processes of covariate identification, VPC implementation, and nonparametric bootstrapping for model stability assessment were performed using the Perl-speaks-NONMEM tool (version 5.3.1, https://uupharmacometrics.github.io/PsN/, accessed on 13 April 2023). Post-analysis processing and graphical representations were performed using the R programming environment (version 4.4.0, https://www.r-project.org/, accessed on 6 June 2024).

### 4.4. Dosage Simulation

To evaluate the appropriateness of current FDA and EMA dosing recommendations, a Monte Carlo simulation was conducted using the population PK model previously developed from data obtained from healthy adult subjects. A total of 20,000 virtual subjects (10,000 female, 10,000 male) were generated, whose demographic characteristics (serum creatinine, age, height, weight, and LBM) were simulated from normal distributions representative of the general adult population. Renal function for each virtual subject was calculated as the eGFR using the CKD-EPI equation, adjusted to individual body surface area (BSA). These simulated subjects were assumed to possess normal renal function. Each virtual subject received piperacillin/tazobactam at a fixed dose of 4 g/0.5 g intravenously q6h, with infusion durations of 0.5, 1, 2, 3, or 4 h. The PTA was calculated based on pathogen-specific PD targets: 50%*f*T_>MIC_ for *P. aeruginosa*, 77%*f*T_>MIC_ for *E. coli*, and 100%*f*T_>MIC_ for *K. pneumoniae*. MIC distributions for these pathogens were sourced from the EUCAST database [16].

Using data from the first simulation, CFR was calculated to assess the likelihood of target attainment across various infusion strategies. The CFR represents the proportion of a simulated population expected to achieve a predefined PD target, based on MIC distributions of the relevant pathogens [11]. CFR was calculated for each pathogen by integrating the PTA across the MIC distribution, using the following formula:

$$CFR=\;\sum_{i}\left({PTA}_{i}\;\times \;f_{i}\right)$$

where *PTA_i_* is the *PTA* at MIC level *i*, and *fi* is the relative frequency of that MIC value in the corresponding pathogen’s distribution. MIC distributions were obtained from the EUCAST database. CFR values were stratified by infusion duration (0.5–4 h) and compared across pathogens to assess the impact of extended infusion on population-level pharmacodynamic outcomes.

In a second simulation study, targeted dosing regimens were evaluated across five distinct renal function categories (eGFR: 0–20, 20–40, 40–90, 90–130, and 130–180 mL/min) in 1000 additional virtual subjects. Piperacillin clearance was calculated using the population PK model. The simulation explored intermittent infusion regimens (doses: 2 g, 4 g, 6 g; intervals: 6 h, 8 h, 12 h; infusion durations: 0.5 h and 3 h) and continuous infusion regimens (daily doses ranging from 4 g to 24 g). Pharmacodynamic targets evaluated were 50%*f*T_>MIC_, 50%*f*T_>4MIC_, 100%*f*T_>MIC_, and 100%*f*T_>4MIC_ across MIC values from 0.5 to 128 mg/L. To specifically evaluate the impact of renal function on dosing, LBM and total body weight were fixed at their median values derived from the original study population.

## Figures and Tables

**Table 1:** Characteristics of the participants.

Parameters	Total (*n* = 12)	Female (*n* = 4)	Male (*n* = 8)
Demographic characteristics			
Age, years	36.0 (26.0–50.0)	29.5 (26.0–36.0)	39.0 (32.0–50.0)
Height, cm	168 (158–182)	163 (158–167)	171 (160–182)
Weight, kg	61.7 (45.8–88.5)	56.1 (45.8–59.5)	69.1 (54.5–88.5)
Lean body mass, kg	50.1 (36.6–65.9)	42.6 (36.6–44.8)	55.2 (45.1–65.9)
Body surface area, m^2^	1.71 (1.44–2.07)	1.60 (1.44–1.66)	1.81 (1.56–2.07)
Body mass index, kg/m^2^	21.5 (18.3–29.7)	21.0 (18.3–21.5)	23.5 (21.3–29.7)
Laboratory characteristics			
Protein, g/dL	7.45 (7.00–8.30)	7.45 (7.30–8.30)	7.45 (7.00–7.70)
Albumin, g/dL	4.80 (4.60–5.20)	4.90 (4.70–5.20)	4.80 (4.60–5.20)
Cystatin C, mg/dL	0.765 (0.620–1.01)	0.700 (0.620–0.740)	0.830 (0.660–1.01)
Creatinine, mg/dL	0.860 (0.590–1.08)	0.690 (0.590–0.750)	0.965 (0.800–1.08)
Blood urea nitrogen, mg/dL	14.1 (9.70–23.0)	12.2 (9.70–16.9)	14.7 (10.5–23.0)
Alanine aminotransferase, U/L	17.0 (3.00–74.0)	10.5 (3.00–15.0)	20.0 (10.0–74.0)
Aspartate aminotransferase, U/L	21.0 (17.0–50.0)	20.0 (17.0–21.0)	24.0 (17.0–50.0)
Gamma-glutamyl transferase, U/L	17.0 (9.00–69.0)	12.5 (9.00–37.0)	26.0 (15.0–69.0)
Renal functions			
CrCl (mL/min) ^a^	105 (76.2–146)	105 (85.0–122)	106 (76.2–146)
BSA adjusted eGFR_MDRD_ (mL/min) ^b^	93.2 (73.3–120)	90.5 (81.5–111)	95.4 (73.3–120)
BSA adjusted eGFR_CKD-EPI_CR_ (mL/min) ^c^	108 (86.2–136)	108 (98.9–115)	110 (86.2–136)
BSA adjusted eGFR_CKD-EPI_CC_ (mL/min) ^d^	110 (86.2–145)	108 (104–112)	113 (86.2–145)
BSA adjusted eGFR_CKD-EPI_CRCC_ (mL/min) ^e^	111 (89.6–145)	110 (104–118)	116 (89.6–145)

CrCl, creatinine clearance by Cockcroft-Gault equation; eGFR, estimated glomerular filtration rate; MDRD, modification of diet in renal disease; CKD-EPI, chronic kidney disease epidemiology collaboration; CR, creatinine; CC, cystatin C; min, the minimum of (CR or CC)/number and 1; max, the maximum of (CR or CC)/number and 1. ^a^ CrCl = (140 − Age) × weight/CR × 72 (×0.85 if female); ^b^ eGFR = 175 × CR^−1.154^ × Age^−0.203^ (×0.742 if female); ^c^ eGFR (female) = 142 × min (CR/0.7,1)^−0.241^ × max (CR/0.7,1)^−1.200^ × 0.9938^Age^ × 1.012/1.73 × BSA; eGFR (male) = 142 × min (CR/0.9,1)^−0.302^ × max (CR/0.9,1)^−1.200^ × 0.9938^Age^ /1.73 × BSA; ^d^ eGFR = 133 × min (CC/0.8,1)^−0.499^ × max (CC/0.8,1)^−1.328^ × 0.9962^Age^ × 0.932 [if female]/1.73 × BSA; ^e^ eGFR (female) = 135 × min (CR/0.7,1)^−0.219^ × max (CR/0.7,1)^−0.544^ × min (CC/0.8,1)^0.323^ × max (CC/0.8,1)^−0.778^ × 0.9961^Age^ × 0.963 /1.73 × BSA; eGFR (male) = 135 × min (CR/0.9,1)^−0.144^ × max (CR/0.9,1)^−0.544^ × min (CC/0.8,1)^0.323^ × max (CC/0.8,1)^−0.778^ × 0.9961^Age^/1.73 × BSA.

**Table 2:** Parameter estimates and bootstrap medians (95% confidence intervals) for the final pharmacokinetic model of piperacillin in 12 healthy adults.

Parameter	Estimates	RSE (%) [Shrinkage, %]	Bootstrap Median (95% CI)
Structural model			
CL = θ_1_ × (CE /108.25) ^θ2^			
θ_1_ (L/h)	11.2	3.40	11.2 (10.5–12.1)
θ_2_	1.16	13.1	1.15 (0.811–1.59)
V1 = θ_3_			
θ_3_ (L)	6.24	8.99	6.19 (5.27–7.57)
Q = θ_4_ × (LBM/50.08) ^θ5^			
θ_4_ (L/h)	4.32 ^a^		
θ_5_	2.50	13.9	2.45 (1.39–3.56)
V2 = θ_6_ × exp (θ_7_ × (WT − 61.7))			
θ_6_ (L)	2.59	3.11	2.59 (2.28–2.74)
θ_7_	0.0288	8.38	0.0284 (0.0208–0.0371)
Interindividual variability			
CL (%)	7.17	30.3 [18.7]	6.08 (0–10.5)
V1 (%)	18.4	28.7 [19.1]	17.5 (0–29.9)
Residual variability			
Proportional error (%)	13.4	12.2 [9.48]	13.1 (9.39–16.0)

RSE, relative standard error; CI, confidence interval; CL, total clearance; V1, central volume of distribution; V2, volume of distribution for the first peripheral compartment; Q, intercompartmental clearance between V1 and V2; CECR, estimated glomerular filtration rate by the CKD-EPI equation, utilizes serum creatinine levels; LBM, lean body mass; WT. weight; ^a^, fixed.

**Table 3:** Parameter estimates and bootstrap medians (95% confidence intervals) for the final pharmacokinetic model of tazobactam in 12 healthy adults.

Parameter	Estimates	RSE (%) [Shrinkage, %]	Bootstrap Median (95% CI)
Structural model			
CL = θ_1_ × (CE /108.25) ^θ2^			
θ_1_ (L/h)	12.4	3.26	12.3 (11.6–13.3)
θ_2_	0.857	13.1	0.858 (0.602–1.21)
V1 = θ_3_			
θ_3_ (L)	9.03	6.40	9.02 (8.05–10.4)
Q = θ_4_			
θ_4_ (L/h)	4.39 ^a^		
V2 = θ_5_ × exp (θ_6_ × (WT − 61.7))			
θ_5_ (L)	3.21	5.48	3.23 (2.68–3.48)
θ_6_	0.0145	16.9	0.0142 (0.0106–0.0238)
Interindividual variability			
CL (%)	6.95	29.0 [7.37]	6.17 (0.403–9.94)
Residual variability			
Proportional error (%)	13.5	9.57 [6.06]	13.2 (10.4–15.6)

RSE, relative standard error; CI, confidence interval; CL, total clearance; V1, central volume of distribution; V2, volume of distribution for the first peripheral compartment; Q, intercompartmental clearance between V1 and V2; CECR, estimated glomerular filtration rate by the CKD-EPI equation, utilizes serum creatinine levels; WT. weight; ^a^, fixed.

## Data Availability

In accordance with institutional policies and to ensure participant confidentiality, the datasets generated and/or analyzed in this study cannot be shared.

## Abbreviations

The following abbreviations are used in this manuscript:

ARC	augmented renal clearance
BSA	body surface area
CC	cystatin C
CFR	cumulative fraction of response
CI	confidence interval
CKD-EPI	chronic kidney disease epidemiology collaboration
CL	total clearance
CR	creatinine
CrCl	creatinine clearance
CWRES	conditional weighted residuals
EDTA	ethylenediaminetetraacetic acid
eGFR	estimated glomerular filtration rate
EMA	European Medicines Agency
EUCAST	European Committee on Antimicrobial Susceptibility Testing
FDA	United States Food and Drug Administration
*f*T_>MIC_	total percentage of a 24 h period during which the concentration of the free (unbound to protein) drug surpasses the minimum inhibitory concentration under steady-state conditions
IIV	interindividual variability
IPRED	individual predictions
IRB	institutional review board
LBM	lean body mass
LC-MS/MS	liquid chromatography-tandem mass spectrometry
MDRD	modification of diet in renal disease
MIC	minimum inhibitory concentration
OFV	objective function value
PK/PD	pharmacokinetic/pharmacodynamic
PRED	population predictions
PTA	probability of target attainment
Q	intercompartmental clearance between V1 and V2
RSE	relative standard error
V1	volume of distribution for the central compartment
V2	volume of distribution for the peripheral compartments
Vd	volume of distribution
VPC	visual predictive check
Vss	steady-state volume of distribution
WT	weight

## Appendix A

**Table A1:** Stepwise covariate selection process of piperacillin: forward selection (*p*-value = 0.01, OFV difference < −6.635, degree of freedom = 1) and backward elimination (*p*-value = 0.001, OFV difference < 10.83, degree of freedom = 1). For comparisons between models using the same covariate but different functional forms (e.g., power vs. exponential), a degree of freedom of 0 was assumed, and a reduction in OFV > 0 was considered statistically significant.

Parameter	Covariate	Model	Base OFV	New OFV	ΔOFV	*p*-Value
Forward step 1						
CL	CrCl ^a^	Power	174.727	160.469	−14.258	0.000159
CL	eGFR, CKD-EPI_MDRD_ ^b^	Power	174.727	161.245	−13.482	0.000241
**CL**	**eGFR, CKD-EPI_CR_ ^c^**	**Power**	**174.727**	**158.832**	**−15.895**	**0.000067**
CL	eGFR, CKD-EPI_CC_ ^d^	Power	174.727	163.464	−11.263	0.000791
CL	eGFR, CKD-EPI_CR-CC_ ^e^	Power	174.727	160.766	−13.961	0.000187
V2	Body mass index	Power	174.727	160.808	−13.919	0.000191
V2	Body surface area	Power	174.727	159.964	−14.763	0.000122
V2	Height	Power	174.727	164.425	−10.302	0.001329
V2	Weight	Power	174.727	164.619	−10.108	0.001476
V2	Body mass index	Power	174.727	161.257	−13.470	0.000242
V2	Weight	Power	174.727	159.464	−15.263	0.000094
Forward step 2						
V2	Body mass index	Power	158.832	145.594	−13.238	0.000274
V2	Body surface area	Power	158.832	144.621	−14.211	0.000163
V2	Height	Power	158.832	149.630	−9.202	0.002418
V2	Weight	Power	158.832	150.497	−8.335	0.003890
V2	Lean body mass	Power	158.832	146.621	−12.211	0.000475
**V2**	**Weight**	**Power**	**158.832**	**144.107**	**−14.725**	**0.000124**
Forward step 3						
Q	Body surface area	Power	144.107	131.553	−12.554	0.000395
Q	Lean body mass	Power	144.107	128.937	−15.170	0.000098
Q	Weight	Power	144.107	132.535	−11.572	0.000670
**V2**	**Weight**	**Exponential**	**144.107**	**143.488**	**−0.619**	**0.000000**
Forward step 4						
Q	Body surface area	Power	143.488	130.285	−13.203	0.000280
**Q**	**Lean body mass**	**Power**	**143.488**	**128.690**	**−14.798**	**0.000120**
Q	Weight	Power	143.488	130.687	−12.801	0.000346
Backward step 1						
CL	eGFR, CKD-EPI_CR_^c^	None	128.690	145.105	16.414	0.000051
Q	Lean body mass	None	128.690	143.488	14.798	0.000120
V2	Weight	Power	128.690	128.937	0.246	0.000000

OFV, objective function value; ΔOFV = New OFV − Base OFV; CrCl, creatinine clearance by Cockcroft-Gault equation; eGFR, estimated glomerular filtration rate; MDRD, modification of diet in renal disease; CKD-EPI, chronic kidney disease epidemiology collaboration; CR, creatinine; CC, cystatin C; min, the minimum of (CR or CC)/number and 1; max, the maximum of (CR or CC)/number and 1. ^a^ CrCl = (140 − Age) × weight/CR × 72 (×0.85 if female); ^b^ eGFR = 175 × CR^−1.154^ × Age^−0.203^ (×0.742 if female); ^c^ eGFR (female) = 142 × min (CR/0.7,1)^−0.241^ × max (CR/0.7,1)^−1.200^ × 0.9938^Age^ × 1.012/1.73 × BSA; eGFR (male) = 142 × min (CR/0.9,1)^−0.302^ × max (CR/0.9,1)^−1.200^ × 0.9938 ^Age^/1.73 × BSA; ^d^ eGFR = 133 × min (CC/0.8,1)^−0.499^ × max (CC/0.8,1)^−1.328^ × 0.9962^Age^ × 0.932 [if female]/1.73 × BSA; ^e^ eGFR (female) = 135 × min (CR/0.7,1)^−0.219^ × max (CR/0.7,1)^−0.544^ × min (CC/0.8,1)^0.323^ × max (CC/0.8,1)^−0.778^ × 0.9961^Age^ × 0.963 /1.73 × BSA.; eGFR (male) = 135 × min (CR/0.9,1)^−0.144^ × max (CR/0.9,1)^−0.544^ × min (CC/0.8,1)^0.323^ × max (CC/0.8,1)^−0.778^ × 0.9961^Age^/1.73 × BSA. Values in red and bold indicate the covariate that was selected in each step of the stepwise analysis.

**Table A2:** Stepwise covariate selection process of tazobactam: forward selection (*p*-value = 0.01, OFV difference < −6.635, degree of freedom = 1) and backward elimination (*p*-value = 0.001, OFV difference < 10.83, degree of freedom = 1). For comparisons between models using the same covariate but different functional forms (e.g., power vs. exponential), a degree of freedom of 0 was assumed, and a reduction in OFV > 0 was considered statistically significant.

Parameter	Covariate	Model	Base OFV	New OFV	ΔOFV	*p*-Value
Forward step 1						
CL	CrCl ^a^	Power	−157.083	−168.790	−11.707	0.000623
CL	eGFR, CKD-EPI_MDRD_ ^b^	Power	−157.083	−167.523	−10.440	0.001233
**CL**	**eGFR, CKD-EPI_CR_ ^c^**	**Power**	**−157.083**	**−168.795**	**−11.712**	**0.000621**
CL	eGFR, CKD-EPI_CC_ ^d^	Power	−157.083	−165.252	−8.169	0.004260
CL	eGFR, CKD-EPI_CR-CC_ ^e^	Power	−157.083	−166.989	−9.906	0.001647
CL	Weight	Power	−157.083	−164.717	−7.634	0.005727
CL	Body surface area	Power	−157.083	−164.675	−7.592	0.005861
V1	Height	Power	−157.083	−164.183	−7.100	0.007710
V2	Body mass index	Power	−157.083	−167.052	−9.969	0.001592
V2	Body surface area	Power	−157.083	−167.924	−10.841	0.000993
V2	Lean body mass	Power	−157.083	−168.305	−11.222	0.000808
V2	Weight	Power	−157.083	−168.610	−11.527	0.000686
Forward step 2						
V1	Height	Power	−168.795	−176.628	−7.833	0.005129
V2	Body mass index	Power	−168.795	−179.349	−10.554	0.001159
V2	Body surface area	Power	−168.795	−181.942	−13.147	0.000288
V2	Height	Power	−168.795	−176.106	−7.312	0.006850
V2	Lean body mass	Power	−168.795	−181.863	−13.068	0.000300
**V2**	**Weight**	**Power**	**−168.795**	**−182.092**	**−13.297**	**0.000266**
Forward step 3						
Q	Body surface area	Power	−182.092	−192.224	−10.132	0.001457
Q	Lean body mass	Power	−182.092	−191.539	−9.447	0.002115
Q	Weight	Power	−182.092	−191.719	−9.627	0.001917
**V2**	**Weight**	**Exponential**	**−182.092**	**−182.631**	**−0.539**	**0.000000**
Forward step 4						
**Q**	**Body surface area**	**Power**	**−182.631**	**−192.957**	**−10.326**	**0.001312**
Q	Lean body mass	Power	−182.631	−191.840	−9.209	0.002409
Q	Weight	Power	−182.631	−192.795	−10.164	0.001432
V1	Height	Power	−182.631	−189.746	−7.115	0.007645
Backward step 1						
CL	eGFR, CKD-EPI_CR_ ^c^	None	−192.957	−179.223	13.733	0.000211
**Q**	**Body surface area**	**None**	**−192.957**	**−182.631**	**10.326**	**0.001312**
V2	Weight	Power	−192.957	−192.224	0.733	0.000000
Backward step 2						
CL	eGFR, CKD-EPI_CR_ ^c^	None	−182.631	−169.313	13.318	0.000263
V2	Weight	Power	−182.631	−182.092	0.539	0.000000

OFV, objective function value; ΔOFV = New OFV − Base OFV; CrCl, creatinine clearance by Cockcroft-Gault equation; eGFR, estimated glomerular filtration rate; MDRD, modification of diet in renal disease; CKD-EPI, chronic kidney disease epidemiology collaboration; CR, creatinine; CC, cystatin C; min, the minimum of (CR or CC)/number and 1; max, the maximum of (CR or CC)/number and 1. ^a^ CrCl = (140 − Age) × weight/CR × 72 (×0.85 if female); ^b^ eGFR = 175 × CR^−1.154^ × Age^−0.203^ (×0.742 if female); ^c^ eGFR (female) = 142 × min (CR/0.7,1)^−0.241^ × max (CR/0.7,1)^−1.200^ × 0.9938^Age^ × 1.012/1.73 × BSA; eGFR (male) = 142 × min (CR/0.9,1)^−0.302^ × max (CR/0.9,1)^−1.200^ × 0.9938 ^Age^/1.73 × BSA; ^d^ eGFR = 133 × min (CC/0.8,1)^−0.499^ × max (CC/0.8,1)^−1.328^ × 0.9962^Age^ × 0.932 [if female]/1.73 × BSA; ^e^ eGFR (female) = 135 × min (CR/0.7,1)^−0.219^ × max (CR/0.7,1)^−0.544^ × min (CC/0.8,1)^0.323^ × max (CC/0.8,1)^−0.778^ × 0.9961^Age^ × 0.963 /1.73 × BSA; eGFR (male) = 135 × min (CR/0.9,1)^−0.144^ × max (CR/0.9,1)^−0.544^ × min (CC /0.8,1)^0.323^ × max (CC /0.8,1)^−0.778^ × 0.9961^Age^/1.73 × BSA. Values in red and bold indicate the covariate that was selected in each step of the stepwise analysis.

**Table A3:** Recommended intermittent piperacillin/tazobactam dosing regimens (probability of target attainment, PTA) to achieve ≥90% PTA for the 50%*f*T_>MIC_ or 100%*f*T_>MIC_ targets, stratified by renal function category (eGFR) and MIC value.

MIC	Target: 50%*f*T_>MIC_		Target: 100%*f*T_>MIC_		
	0.5 h Infusion	3 h Infusion	0.5 h Infusion	3 h Infusion	CI
**BSA adjusted eGFR: 0–20 mL/min**					
0.5	2 g q12h (100)	2 g q12h (100)	2 g q12h (100)	2 g q12h (100)	4 g (100)
1	2 g q12h (100)	2 g q12h (100)	2 g q12h (100)	2 g q12h (100)	4 g (100)
2	2 g q12h (100)	2 g q12h (100)	2 g q12h (100)	2 g q12h (100)	4 g (100)
4	2 g q12h (100)	2 g q12h (100)	2 g q12h (100)	2 g q12h (100)	4 g (100)
8	2 g q12h (100)	2 g q12h (100)	2 g q12h (100)	2 g q12h (100)	4 g (100)
16	2 g q12h (100)	2 g q12h (100)	2 g q12h (98.3)	2 g q12h (100)	4 g (100)
32	2 g q12h (100)	2 g q12h (100)	2 g q8h (100)	2 g q12h (91.5)	4 g (100)
64	2 g q12h (94.1)	2 g q12h (96.6)	2 g q6h (100)	2 g q8h (94.1)	4 g (100)
128	2 g q6h (96.6)	2 g q6h (100)	4 g q6h (100)	4 g q8h (94.1)	8 g (100)
**BSA adjusted eGFR: 20–40 mL/min**					
0.5	2 g q12h (100)	2 g q12h (100)	2 g q12h (100)	2 g q12h (100)	4 g (100)
1	2 g q12h (100)	2 g q12h (100)	2 g q12h (100)	2 g q12h (100)	4 g (100)
2	2 g q12h (100)	2 g q12h (100)	2 g q12h (94.6)	2 g q12h (100)	4 g (100)
4	2 g q12h (100)	2 g q12h (100)	2 g q8h (100)	2 g q8h (100)	4 g (100)
8	2 g q12h (100)	2 g q12h (100)	2 g q8h (96.8)	2 g q8h (100)	4 g (100)
16	2 g q12h (98.9)	2 g q12h (100)	2 g q6h (100)	2 g q6h (100)	4 g (100)
32	2 g q8h (100)	2 g q8h (100)	4 g q6h (100)	2 g q6h (95.7)	4 g (100)
64	4 g q8h (100)	2 g q6h (100)	6 g q6h (89.2)	4 g q6h (95.7)	8 g (100)
128	6 g q6h (100)	4 g q6h (100)	6 g q6h (40.9)	6 g q6h (66.7)	16 g (100)
**BSA adjusted eGFR: 40–90 mL/min**					
0.5	2 g q12h (99.3)	2 g q12h (100)	2 g q6h (99.3)	2 g q8h (97.2)	4 g (100)
1	2 g q12h (91.5)	2 g q12h (100)	2 g q6h (91.5)	2 g q6h (100)	4 g (100)
2	2 g q8h (100)	2 g q12h (99.3)	4 g q6h (91.5)	2 g q6h (99.3)	4 g (100)
4	2 g q8h (96.8)	2 g q8h (100)	6 g q6h (83.1)	4 g q6h (99.3)	4 g (100)
8	2 g q6h (98.9)	2 g q8h (100)	6 g q6h (64.8)	6 g q6h (97.2)	4 g (100)
16	4 g q6h (98.9)	2 g q6h (100)	6 g q6h (44.0)	6 g q6h (75.4)	8 g (100)
32	6 g q6h (91.2)	4 g q6h (100)	6 g q6h (20.1)	6 g q6h (46.1)	12 g (100)
64	6 g q6h (54.6)	6 g q6h (100)	–	6 g q6h (17.6)	20 g (96.1)
128	6 g q6h (14.8)	6 g q6h (42.6)	–	–	24 g (42.6)
**BSA adjusted eGFR: 90–130 mL/min**					
0.5	2 g q8h (100)	2 g q12h (98.7)	6 g q6h (83.6)	2 g q6h (98.7)	4 g (100)
1	2 g q8h (93.5)	2 g q8h (100)	6 g q6h (45.7)	4 g q6h (98.7)	4 g (100)
2	2 g q6h (100)	2 g q8h (100)	6 g q6h (19.0)	6 g q6h (93.5)	4 g (100)
4	4 g q6h (100)	2 g q8h (100)	–	6 g q6h (53.0)	4 g (100)
8	6 g q6h (96.1)	2 g q6h (100)	–	6 g q6h (15.5)	4 g (98.7)
16	6 g q6h (51.3)	2 g q6h (98.7)	–	–	8 g (98.7)
32	–	4 g q6h (98.7)	–	–	16 g (98.7)
64	–	6 g q6h (40.9)	–	–	24 g (39.2)
128	–	–	–	–	–
**BSA adjusted eGFR: 130–180 mL/min**					
0.5	2 g q6h (100)	2 g q8h (100)	–	6 g q6h (93.4)	4 g (100)
1	4 g q6h (100)	2 g q8h (100)	–	6 g q6h (52.7)	4 g (100)
2	6 g q6h (99.6)	2 g q8h (96.3)	–	6 g q6h (14.3)	4 g (100)
4	6 g q6h (69.6)	2 g q6h (100)	–	–	4 g (100)
8	6 g q6h (20.1)	2 g q6h (100)	–	–	8 g (100)
16	–	4 g q6h (100)	–	–	12 g (98.2)
32	–	6 g q6h (98.2)	–	–	24 g (98.2)
64	–	–	–	–	–
128	–	–	–	–	–

CI, continuous infusion; BSA, body surface area. Shaded cells indicate dosing regimens that achieved ≥90% PTA.

**Table A4:** Recommended intermittent piperacillin/tazobactam dosing regimens (probability of target attainment, PTA) to achieve ≥90% PTA for the 50%*f*T4_>MIC_ or 100%*f*T_>4MIC_ targets, stratified by renal function category (eGFR) and MIC value.

MIC	Target: 50%*f*T_>4MIC_		Target: 100%*f*T_>4MIC_		
	0.5 h Infusion	3 h Infusion	0.5 h Infusion	3 h Infusion	CI
**BSA adjusted eGFR: 0–20 mL/min**					
0.5	2 g q12h (100)	2 g q12h (100)	2 g q12h (100)	2 g q12h (100)	4 g (100)
1	2 g q12h (100)	2 g q12h (100)	2 g q12h (100)	2 g q12h (100)	4 g (100)
2	2 g q12h (100)	2 g q12h (100)	2 g q12h (100)	2 g q12h (100)	4 g (100)
4	2 g q12h (100)	2 g q12h (100)	2 g q12h (98.3)	2 g q12h (100)	4 g (100)
8	2 g q12h (100)	2 g q12h (100)	2 g q8h (100)	2 g q12h (91.5)	4 g (100)
16	2 g q12h (94.1)	2 g q12h (96.6)	2 g q6h (100)	2 g q8h (94.1)	4 g (100)
32	2 g q6h (96.6)	2 g q6h (100)	4 g q6h (100)	4 g q8h (94.1)	8 g (100)
64	4 g q6h (96.6)	4 g q6h (100)	6 g q6h (94.1)	6 g q6h (100)	16 g (100)
128	6 g q6h (80.5)	6 g q6h (88.1)	6 g q6h (63.6)	6 g q6h (71.2)	24 g (88.1)
**BSA adjusted eGFR: 20–40 mL/min**					
0.5	2 g q12h (100)	2 g q12h (100)	2 g q12h (94.6)	2 g q12h (100)	4 g (100)
1	2 g q12h (100)	2 g q12h (100)	2 g q8h (100)	2 g q8h (100)	4 g (100)
2	2 g q12h (100)	2 g q12h (100)	2 g q8h (96.8)	2 g q8h (100)	4 g (100)
4	2 g q12h (98.9)	2 g q12h (100)	2 g q6h (100)	2 g q6h (100)	4 g (100)
8	2 g q8h (100)	2 g q8h (100)	4 g q6h (100)	2 g q6h (95.7)	4 g (100)
16	4 g q8h (100)	2 g q6h (100)	6 g q6h (89.2)	4 g q6h (95.7)	8 g (100)
32	6 g q6h (100)	4 g q6h (100)	6 g q6h (40.9)	6 g q6h (66.7)	16 g (100)
64	6 g q6h (44.1)	6 g q6h (58.1)	–	6 g q6h (23.7)	24 g (58.1)
128	–	–	–	–	–
**BSA adjusted eGFR: 40–90 mL/min**					
0.5	2 g q8h (100)	2 g q12h (99.3)	4 g q6h (91.5)	2 g q6h (99.3)	4 g (100)
1	2 g q8h (96.8)	2 g q8h (100)	6 g q6h (83.1)	4 g q6h (99.3)	4 g (100)
2	2 g q6h (98.9)	2 g q8h (100)	6 g q6h (64.8)	6 g q6h (97.2)	4 g (100)
4	4 g q6h (98.9)	2 g q6h (100)	6 g q6h (44.0)	6 g q6h (75.4)	8 g (100)
8	6 g q6h (91.2)	4 g q6h (100)	6 g q6h (20.1)	6 g q6h (46.1)	12 g (100)
16	6 g q6h (54.6)	6 g q6h (100)	–	6 g q6h (17.6)	20 g (96.1)
32	6 g q6h (14.8)	6 g q6h (42.6)	–	–	24 g (42.6)
64	–	–	–	–	–
128	–	–	–	–	–
**BSA adjusted eGFR: 90–130 mL/min**					
0.5	2 g q6h (100)	2 g q8h (100)	6 g q6h (19)	6 g q6h (93.5)	4 g (100)
1	4 g q6h (100)	2 g q8h (100)	–	6 g q6h (53.0)	4 g (100)
2	6 g q6h (96.1)	2 g q6h (100)	–	6 g q6h (15.5)	4 g (98.7)
4	6 g q6h (51.3)	2 g q6h (98.7)	–	–	8 g (98.7)
8	–	4 g q6h (98.7)	–	–	16 g (98.7)
16	–	6 g q6h (40.9)	–	–	24 g (39.2)
32	–	–	–	–	–
64	–	–	–	–	–
128	–	–	–	–	–
**BSA adjusted eGFR: 130–180 mL/min**					
0.5	6 g q6h (99.6)	2 g q8h (96.3)	–	6 g q6h (14.3)	4 g (100)
1	6 g q6h (69.6)	2 g q6h (100)	–	–	4 g (100)
2	6 g q6h (20.1)	2 g q6h (100)	–	–	8 g (100)
4	–	4 g q6h (100)	–	–	12 g (98.2)
8	–	6 g q6h (98.2)	–	–	24 g (98.2)
16	–	–	–	–	–
32	–	–	–	–	–
64	–	–	–	–	–
128	–	–	–	–	–

CI, continuous infusion; BSA, body surface area. Shaded cells indicate dosing regimens that achieved ≥90% PTA.
